# Swedish high school students' knowledge and attitudes regarding fertility and family building

**DOI:** 10.1186/1742-4755-9-6

**Published:** 2012-03-21

**Authors:** Maria Ekelin, Cecilia Åkesson, Malin Ångerud, Linda J Kvist

**Affiliations:** 1 Department of Health Sciences, Division of Nursing, Lund University, P.O Box 157 SE-221 00 LUND, Sweden; 2 Department of Obstetrics and Gynaecology, Kristianstad Hospital, Kristianstad, Sweden; 3 Department of Obstetrics and Gynaecology, Helsingborg Hospital, Helsingborg, Sweden

**Keywords:** Fertility, Infertility, High school students, Questionnaire

## Abstract

**Background:**

Infertility is a serious problem for those who suffer. Some of the risks for infertility are preventable and the individual should therefore have knowledge of them. The purposes of this study were to investigate high-school students' knowledge about fertility, plans for family building and to compare views and knowledge between female and male students.

**Methods:**

A questionnaire containing 34 items was answered by 274 students. Answers from male and female students were compared using student's *t*-test for normally distributed variables and Mann-Whitney *U*-test for non-normal distributions. The chi-square test was used to compare proportions of male and female students who answered questions on nominal and ordinal scales. Differences were considered as statistically significant at a *p*-value of 0.05.

**Results:**

Analyses showed that 234 (85%) intended to have children. Female students felt parenthood to be significantly more important than male students: *p *= *<*0.01. The mean age at which the respondents thought they would like to start to build their family was 26 (± 2.9) years. Men believed that women's fertility declined significantly later than women did: *p *= *<*0.01. Women answered that 30.7% couples were involuntarily infertile and men answered 22.5%: *p *= *<*0.01. Females thought it significantly more likely that they would consider IVF or adoption than men, *p = *0.01. Men felt they were more likely to abstain from having children than women: *p = <*0.01. Women believed that body weight influenced fertility significantly more often than men: *p = <*0.01 and men believed significantly more often that smoking influenced fertility: *p *= 0.03. Both female and male students answered that they would like to have more knowledge about the area of fertility.

**Conclusions:**

Young people plan to start their families when the woman's fertility is already in decline. Improving young people's knowledge about these issues would give them more opportunity to take responsibility for their sexual health and to take an active role in shaping political change to improve conditions for earlier parenthood.

## Background

Infertility is a common problem with an estimated median prevalence of 9% worldwide [1] and 10-20% prevalence in Scandinavia [2]. Since some of the risks for infertility can be prevented, it is of great interest to investigate whether young people around the age for sexual début are aware of these risks, have knowledge about fertility in general and if they have considered their own future family planning. In 1998 a report from Sweden [3] showed that the mean age for sexual début for women was 16.5 years and for men 16.8 years. These figures were seen to be similar in 2002 [4].

Research has shown that most individuals intend to reproduce at some stage in their lives [5-7]. Factors which influence the time chosen for the start of family building are similar in several high income countries and include a suitable partner, economic stability and that both the partner and self feel that the time is appropriate [5,8]. Young women considered that "appropriate timing" for family building would be after they fulfilled their wishes for travel and personal development [9]. A majority of the women in the study had planned a life strategy that allowed family building only after they had established their independence through education and the founding of a career.

The average age for a first childbirth in Sweden has increased by four years during the last 30 years and is now 29 years [10]. Young women's fertility is somewhat reduced already around the age of 20 to 30 years and after 35 years it is reduced increasingly faster and therefore postponing childbirth increases the risk for involuntary infertility [11-13]. Children born to older mothers have an increased occurrence of chromosomal changes [14]. There are several other problems associated with postponed pregnancy including risks for complications during pregnancy and childbirth such as miscarriage [15], diabetes, hypertension, premature birth and labour dystocia [11-13]. Complications of pregnancy and childbirth lead to increased use of hospital and specialist healthcare. Infertility leads often to investigations and in-vitro fertilisation treatments. The economic consequences of these investigations and treatments are felt by both individuals and societies [16,17].

It has been shown that awareness of risks for infertility, such as sexually transmitted infections (STI) is not optimal among young people [18] . In California a study of 302 young people (12-17 years) was carried out in an area with a high prevalence of STI [19]. A total of 58% of the young people in the study did not believe that they could influence their own possible future fertility problems. Only a little more than half of the respondents had knowledge about the association of specific STI and fertility. Findings from Canada [7] where 772 young people participated showed little knowledge regarding the association between Chlamydia, gonorrhoea and infertility. In the school where the study took place sex education was not compulsory. In Sweden, Chlamydia is the most often identified STI and has increased three-fold during the last 10 year period. Of those affected, 88% are aged between 15 and 29 years [20]. These statistics may indicate that there is potential in young people's lives for many sexual contacts before they start to consider building their families.

It has been shown that life-style factors such as high or low BMI, excessive exercising, smoking and use of alcohol all affect fertility negatively [21]. High body mass index (BMI) has shown a statistically significant relationship to male infertility [22]. A study from England suggested that there could be a cumulative effect of negative life-style factors on fertility [23]. According to the authors infertility could be reduced by more than 50% if couples lived a healthy life-style and this, in the long term, could lead to a reduction in investigations and treatments for infertility.

Previously, Swedish midwives had an extensive "out-reach" programme for the teaching of sex education to high-school students. At present, according to the regulation of high-school curriculums, students' education in sexual and reproductive health is the responsibility of the school's principal. Facts about alcohol, tobacco, drugs and sexual and reproductive health are seen as part of the same area of knowledge and are required to be integrated into different taught subjects. A Swedish study has shown that a majority of female high-school students are dissatisfied with the quality of education in sexual and reproductive health given at school [24]. Although university student's knowledge of fertility has earlier been examined in Sweden [6], research regarding high school students' fertility knowledge has been examined to a lesser degree.

The aims of this study were to investigate high-school students' knowledge about fertility in general, their future plans for family building and to compare the views and knowledge between female and male students.

## Methods

The study was carried out in the county of Skåne in Southern Sweden in 2009 using a modified questionnaire earlier developed by Swedish researchers [5]. Skåne, with a population of 1, 250 000, has the third largest population of Swedish counties.

### The instrument

The instrument used in this study was based on a validated questionnaire developed by two midwives, a gynaecologist and a psychologist; validation tests were carried out in three separate pilot studies, which included 60 students [5]. The questionnaire was revised according to test results and comments from students. The original instrument was composed of 56 items contained within seven domains. The domains were; demographic data, intention to have children, importance of having children, behavioural intention in case of infertility, conditions of importance for the decision to become a parent, perceived life changes in connection with becoming a parent and awareness of fertility issues [5]. Permission to use the questionnaire in part and to modify it for its use in the present study was given by the first author, Claudia Lampic. Items from the domains pertaining to conditions of importance for the decision to become a parent and perceived life changes in connection with becoming a parent were not included in our modified version since these domains were not within the scope of this study and the respondents were of an age where these questions were felt to be less pertinent.

The revised version consisted of 34 items, 17 of which were taken from the original instrument. Five items were demographic variables: gender, age, name of study program, whether born in Sweden or not and religious belief (three items from the original questionnaire). The remaining 29 items were as follows: intention to have children (four items as in the original questionnaire), importance of having children (one item as in the original questionnaire), behavioural intention in case of infertility (three items as in the original questionnaire) and awareness of fertility issues (21 items: six from the original questionnaire and 15 items which were constructed for this study).

To test for face validity the questionnaire was answered by ten young people between 17 and 18 years of age. They stated that they had no difficulties understanding or answering the questions and therefore no changes were made.

### The study population and data collection

The study population consisted of a convenience sample of students aged 18-20 from the final year of secondary education (high school) in a medium sized town with a population of approximately 36,000. The area is urban and the main source of employment is the foodstuffs industry. Two schools participated; one specialising in theoretical courses and one in practical courses. In Sweden young people over the age of 15 years may be approached to fill in questionnaires without parental consent being sought. Written information about the study was given to the students and their willingness to complete the questionnaire was taken to be informed consent. The class teachers gave spoken information about the study to the students and repeated the written information that participation was completely voluntary and that the school had no interest in whether the students filled in the questionnaire or not and that returned questionnaires would not be read by any of the school's employees. Names and contact details of three of the authors were given to the students in case any of them felt that they needed to discuss any of the issues taken up in the questionnaire. The contact details were also given to the teachers in case they noticed that there were any student concerns.

The inclusion criteria were that the students should have had their 18th birthday and had a mastery of the Swedish language. The intention was that all students who fulfilled the criteria and attended classes on a given date should be asked to participate. After completion, the questionnaires were, in the presence of the students, placed directly in a pre-addressed envelope, which was sealed. Two of the authors (MA and CÅ) collected the envelopes from the schools.

### Ethical considerations

The Advisory Committee for Research Ethics in Health Education at Lund University gave an advisory statement about the study (DR 75-10). We do not know whether the questionnaire might have been of a sensitive nature to some of the respondents. However, participation was optional and it is therefore possible that those students who found the area of investigation too sensitive decided not to answer. Despite written and oral information about inclusion criteria, 19 of the students who completed the questionnaire had not had their 18th birthday. It was decided to include these questionnaires in the analysis since there was no legal compulsion to discard them and the students expressed, by filling in the questionnaire, a desire to partake.

### Analyses

The material from the questionnaires was analysed using SPSS 15.0 and descriptive and analytical statistics were used. The answers of the male and female students were compared using student's *t*-test for normally distributed continuous variables and Mann-Whitney *U*-test for non-normal distributions. The chi-square test was used to compare proportions of male and female students who answered questions on nominal and ordinal scales. Pearson's correlation test was used to test for correlations between the age at which the respondents thought that they would like to start their family and whether they would, in case of infertility, consider IVF. Differences were considered as statistically significant at a *p*-value of 0.05.

## Results

There were 275 students in classes on the day the questionnaire was administered and 247 (90%) chose to participate. Of the 28 questionnaires that were not completed, 22 were due to the fact that the students had not had their 18th birthday. Of the students who completed the questionnaire, 228 (92.3%) were between18 and 20 years of age and 19 (7.7%) were 17 years old. Table 1 shows details of background variables for the study participants.

**Table 1:** Background information about the study participants

Background Variables	*n *(%)
**Gender**	
Female	101 (40.9)
Male	146 (59.1)
	
**Study programs**	
Practical programs	137 (55.4)
Theoretical programs	110 (44.6)
	
**Place of birth**	
Sweden	231 (93.5)
Other	13 (5.3)
Missing	3 (1.2)
	
**Religious conviction**	
Christianity	160 (64.8)
Islam	17 (6.9)
No religious conviction	48 (19.4)
Missing	22 (8.9)

### Intention to have children and importance of parenthood

The analysis showed that 234 (96%) of the respondents wished to become parents in the future. Seven (3%) had no wish to have children and 3 (1%) answered"I don't know". Table 2 shows the number of children that the respondents stated they wished to have in the future and the age at which they thought they would start to build their family. Many women (44%) and a majority of the men (69%) stated that their wish was for two children. Women wished to have significantly more children than men (*p *= < 0.01). In the whole study population the mean age at which the respondents thought they would like to build their family was 26 (± 2.9) years. Men suggested a significantly higher age than women for the start of family building: 26.5 years (± 2.7) vs 25.5 years (± 3.2), *p *= 0.02. There was no significant difference between men and women for the age at which they wished to have their last child: men suggested 32.5 (± 4.4) years and women 32.7 (± 4.4) years.

**Table 2:** Women's and men's answers regarding future plans for family building

Question	Women (*n *101)	Men (*n *146)
	*n *(%)	*n *(%)
**Do you want children in the future?**		
Yes	97 (97)	137 (95.1)
**How many children do you want to have?**		
1	5 (5.4)	5 (3.9)
2	40 (43.5)	89 (69)
3	31 (33.7)	28 (21.7)
4	12 (13.1)	6 (4.7)
5 or more	4 (4.3)	1 (0.8)
**At what age would you like to have your first child?**		
18-21 years	7 (7.7)	4 (3.1)
22-24 years	25 (27.5)	19 (14.8)
25-29 years	50 (55)	85 (65.9)
≥ 30 years	9 (9.9)	21 (16.3)
**At what age would you like to have your last child?**		
23-29 years	20 (22.5)	25 (20.7)
30-34 years	34 (38.1)	53 (43.7)
35-39 years	27 (30.2)	34 (28.1)
≥ 40 years	8 (8.9)	9 (7.4)

A comparison of answers pertaining to the importance of future parenthood showed that female students felt it to be significantly more important than male students (*p *= < 0.01).

### Awareness of fertility issues

Respondents answered on a visual analogue scale (VAS) how important they considered their own fertility to be. The scale ranged from 0 = "not at all important" to 10 "of the greatest importance". The mean score was 7.9 (± 2.2). There was no statistically significant difference between young women and young men concerning how important they considered their own fertility; *p *= 0.06.

In answer to the question regarding when during the menstrual cycle the possibility for a pregnancy was greatest, significantly more men than women answered" after the menstrual bleeding": (Chi ^2 ^= 6.80, *p *= < 0.01) and conversely, significantly more women than men thought that pregnancy was most likely in the middle of the menstrual cycle (Chi ^2 ^= 21.24, *p *= < 0.01). Table 3 shows a statistical comparison of male and female students' answers to questions that measure their awareness of fertility issues. The results show that the students over-estimated the period during which a woman is fertile, the chance/risk of pregnancy and the success rate of in-vitro fertilization. They also over-estimated the percentage of couples who are involuntarily infertile.

**Table 3:** A comparison of men and women's answers to questions regarding fertility

Question	Female respondents Mean (SD)	Male respondents Mean (SD)	*t*-value	*p*-value	Correct answer
At what age is a woman most fertile?	22 yrs (± 5.1)	22 yrs (± 3.8)	0.0	1.0	20-24 yrs
At what age does a woman's fertility start to decline?	37.5 yrs (± 6.2)	37.5 yrs (± 3.8)	0.0	1.0	25-29 yrs
At what age does a woman's fertility decline markedly?	44 yrs (± 6.0)	47.5 yrs (± 10.9)	2.90	**< 0.01**	35-39 yrs
What is the chance/risk (in %) of a pregnancy occurring when a young man and woman under 25 years of age have unprotected intercourse?	72.5% (± 18.6)	64.5% (± 23.5)	-2.7	**< 0.01**	30-39%
What percentage of couples do you estimate are involuntarily infertile?	30.7% (± 20.0)	22.5% (± 16.3)	-3.3	**< 0.01**	10-19%
How often (in %) do you estimate that in-vitro fertilization is successful?	45.2% (± 20.8)	49.5% (± 23.0)	1.40	0.16	20-29%

Student's *t*-test used to test for mean differences between the groups. Correct answers to the questions (Lampic et al., 2005) are given in the right hand column

**Figure 1:**
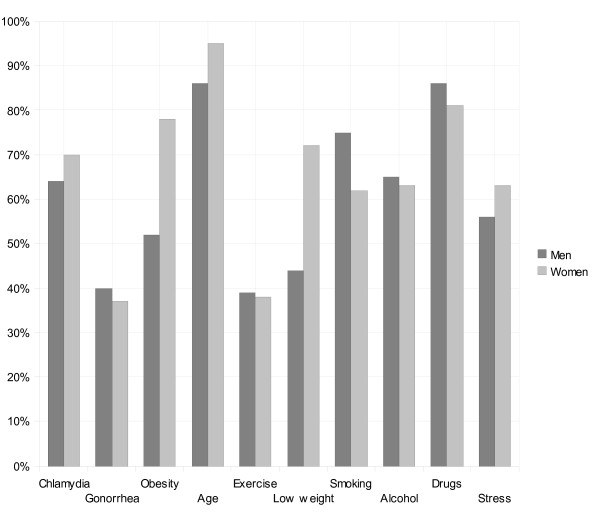
**Percentages of men and women who answered that fertility could be affected by these variables**.

Figure 1 shows proportions of women and men who considered that fertility can be influenced by Chlamydia infection, Gonorrhoea, overweight, age, excessive exercise, underweight, smoking, alcohol consumption, drugs and stress. Women answered to significantly greater extent than men that overweight (Chi ^2 ^= 17.48, *p *= < 0.01), underweight (Chi ^2 ^= 19.55, *p *= < 0.01) and age (Chi ^2 ^= 3.82, *p *= 0.05) were associated with infertility. Men answered significantly more often that smoking (Chi ^2 ^= 4.78, *p *= 0.03) was associated with infertility. For the remaining risk factors there were no differences between women's and men's answers.

On visual analogue scales where 0 indicated "not at all" and 10 indicated "in the highest degree" questions about personal fertility were answered. There were no significant differences between answers given by women and men. Women gave a mean score of 6.0 (± 1.8) for their possibility to influence their own fertility and men gave a mean score of 5.4(± 2.2). They indicated that their knowledge about fertility was less than optimal; women gave a mean score of 4.3 (± 2.4) and men gave a mean score of 4.7 (± 2.9). Both men and women would like to learn more about fertility; women gave a mean score of 6.2 (± 3.1) and men gave a mean score of 6.0 (± 2.8).

### Behavioural intention in case of infertility

Table 4 shows comparisons between men and women for questions about what options they might consider in case of future fertility problems. Women thought it significantly more likely that they would consider in-vitro fertilisation (IVF) or adoption than men did. Men thought that they were more likely to abstain from having children to a significantly higher degree than women. There were no statistically significant correlations between the age at which men or women would like to start their family and their willingness to consider IVF.

**Table 4:** A comparison of mean scores between men and women for answers to hypothetical questions regarding possible future infertility

Question	Women	Men	*p*-value
	Mean (SD)	Mean (SD)	
I would consider artificial insemination (in-vitro fertilisation).	6.6 (± 3.3) *n *= 100	4.3 (± 3.2) *n *= 141	**0.01**
I would consider adoption	6.2 (± 3.0) *n *= 99	3.4 (± 3.0) *n *= 145	**< 0.01**
I would abstain from having children	1.8 (± 2.3) *n *= 95	3.4 (± 3.3) *n *= 142	**< 0.01**

Answers given on a visual analogue scale where 0 = "not at all likely" and 10 = "very likely"

## Discussion

Validity of the original questionnaire cannot be claimed for the present study because of the changes made. However, in the present study, face validity was tested by ten young people who had no difficulties in understanding or answering the questions. It is possible to claim some measure of reliability since the results of the present study were similar to results from a study where the original questionnaire was used for the first time. The low drop-out rate is a strength of the paper although results cannot be generalised to all schools in Sweden.

During analysis of the questionnaires we became aware that the question concerning when during the menstrual cycle the woman is most likely to conceive, may have been misleading. The alternative answers were: "during menstruation", "after menstruation" and "in the middle of the cycle". Since ovulation in fact takes place both "after menstruation" and "in the middle of the cycle" it would have been more correct if the alternative had been worded thus: "directly after menstruation", so that the difference in choice of answers would have been more clear.

The question regarding the chance/risk of a pregnancy when a young couple had unprotected intercourse would have been more correct if the word "once" had been written after the word "intercourse" since repeated intercourse would give an increase in likelihood for a pregnancy.

Although both written and verbal instructions about the intended age of the respondents were given to the students, there were 19 questionnaires that were filled in by students who were less than 18 years of age, which was 7.7% of the respondents. Since there was no judicial problem to include 17-year-old students we decided that the most ethical choice was to include their questionnaires, since they obviously wished to participate.

Both women and men showed a tendency to over-estimate rates of infertility and to overestimate the chance of pregnancy with both unprotected intercourse and IVF. Balasch [25] showed that belief in the infallibility of reproduction technology was widespread and that young people thought that technology could compensate age-related reduction in fertility. Young men in the present study were unclear about when in the menstrual cycle conception was most likely to occur and both men and women felt their knowledge about fertility could be improved. Several factors that affect fertility can be influenced by the individual and improving young people's knowledge in this area may enable them to take conscious decisions regarding their own reproductive health. Some 15 to 20 years ago Swedish midwives took responsibility for the education of high school students in matters of sexual health and their out-reach activities were nation-wide. This work was abandoned due to lack of funding in health care services and the responsibility for sexual education was given to class teachers. It is indeed short-sighted politics for a society to reduce expenditure on sexual education and then foot the bill for infertility treatments.

A majority of the respondents in this study (97% of the women and 95% of the men) wished to have children in the future, which was in accordance with results from an earlier Swedish study [5]. The present study has shown that women set greater value on becoming a parent (either naturally, by adoption or IVF) than men.

As we have already reported [10], the mean age at birth of the first child in Sweden is 29 years for women and 31 years for men. The young women and men in the present study thought themselves likely to start their families earlier (26 years) than these statistics show. Life-style factors may influence the individual's fertility and there is an inherent risk that increasing age together with reduced fertility might mean that they do not have time to have the number of children they wish to have. It may be difficult for young people to envisage the economical pre-requisites for family building or even the availability of a suitable partner. It has been shown that permanent employment, economic stability and a partner suitable as a parent are the most important pre-requisites for family building [8]. Providing suitable housing for a family is dependent, for most people, on a steady income. In the present economic climate it is necessary to have education and experience in order to compete for permanent employment. All of this is time consuming and the biological clock ticks on.

The respondents had realistic ideas about the age at which a woman is most fertile but less realistic ideas about when fertility started to decline and when a tangible decline takes place. These results are in line with research reported by Bunting and Boivin [26] who showed that young people in Wales were aware of the correlation between age and reduced fertility but that despite this, the number of women giving birth after 35 years of age continued to increase during a ten-year period. A consequence of this situation can be that attempts to build families may be left so late that there is a risk that spontaneous pregnancy may not occur expediently and that passing time further reduces fertility. Although costs of assisted human reproduction technologies (ART) have decreased they still represent a substantial cost for welfare states or individuals [17].

In the present study young people thought that they could influence their own fertility to some extent but felt that they needed more knowledge about the factors involved in reduced fertility. It is a serious criticism of the education in sexual health provided for young Swedish people that many were unaware that common sexually transmitted diseases such as Chlamydia and Gonorrhoea could negatively affect fertility. A study from USA showed that young people felt that sex education gave them a greater possibility to take conscious decisions regarding sex, that their sexual début occurred later and that they used contraceptives to a greater degree than those who had not received any formal sex education [27]. Researchers have brought attention to the fact that specific interventions in sexual education programs in schools are problematical because their effects are often insufficiently evaluated and that the interventions therefore cannot yet be widely recommended [28,29]. Generally speaking young people in Sweden today have a liberal view of sexuality. A study carried out by the Swedish National Board for Health and Welfare [20] confirmed the changing attitudes of young people towards sexual activity, which has led to an increase in the number of sexual partners. Trent, Millstein and Ellen [19] called for STI prevention programs aimed at young people not yet sexually active. According to WHO [30] when programs of this kind are coupled with discussions about values they could lead to later sexual début and a wider use of prevention against pregnancy and STI.

School has a central role in the lives of young people and should therefore be used as a platform for education in other areas than the purely academic. We suggest that the results of this study show a need for much improvement in the provision of education in sexual health matters for young people in Sweden. There are both personal and societal implications for improvement in the individual's possibility for future reproduction.

## Conclusions and implications for change

It has been shown that Swedish young people lacked knowledge regarding conception, the success of IVF and factors that influence fertility. This lack of knowledge was recognised by the students who wished to be given more education on the subject of reproduction. Their preference for the age at which they would like to start their families was in fact at an age when the woman's fertility was already on the decline. It is important that young people who are soon to be parents have knowledge that will increase their chances of natural child-bearing.

Educational co-operation between different groups who have contact with young students, for example teachers, youth centres (midwives) and school medical workers, may be a way to improve this area of knowledge. Education should be adapted to the lifestyle and needs of young people and should mirror societal needs. Governments may feel inclined to improve the standard of sex education when they realize the potential for saving expenditure on fertility treatments.

### Further research

The results of the present study indicate that knowledge needs differ between the genders. Further studies could examine whether teaching in matters of sexual health is best carried out in mixed-gender groups or in separate- gender groups. It would also be of interest to carry out longitudinal studies to examine whether the young people in fact had their children as they thought they would and to identify factors that influenced their choices for reproduction.

## Competing interests

The authors declare that they have no competing interests.

## Authors' contributions

CÅ MÅ and LJK planned and designed the study. CÅ and MÅ collected the data. CÅ MÅ LJK and ME were involved in the compilation of the results, drafting and critically assessing the manuscript. All authors read and approved the final manuscript.
